# A deep learning approach for monitoring parietal-dominant Alzheimer’s disease in World Trade Center responders at midlife

**DOI:** 10.1093/braincomms/fcab145

**Published:** 2021-07-02

**Authors:** Allen P F Chen, Sean A P Clouston, Minos Kritikos, Lauren Richmond, Jaymie Meliker, Frank Mann, Stephanie Santiago-Michels, Alison C Pellecchia, Melissa A Carr, Pei-Fen Kuan, Evelyn J Bromet, Benjamin J Luft

**Affiliations:** 1 Medical Scientist Training Program, Department of Neurobiology and Behavior, Renaissance School of Medicine at Stony Brook University, Stony Brook, NY 11794, USA; 2 Family, Population, and Preventive Medicine, Renaissance School of Medicine at Stony, Brook University, Stony Brook, NY 11794, USA; 3 Program in Public Health, Renaissance School of Medicine at Stony, Brook University, Stony Brook, NY 11794, USA; 4 Department of Psychology, Stony Brook University, Stony Brook, NY 11794, USA; 5 Stony Brook World Trade Center Wellness Program, Renaissance School of Medicine at Stony, Brook University, Stony Brook, NY 11725, USA; 6 Department of Applied Mathematics and Statistics, Stony Brook University, Stony Brook, NY 11794, USA; 7 Department of Psychiatry, Renaissance School of Medicine at Stony Brook University, Stony Brook, NY 11794, USA; 8 Department of Medicine, Renaissance School of Medicine at Stony Brook University, Stony Brook, NY 11794, USA

**Keywords:** cognitive impairment, World Trade Center, artificial neural network, parietal-dominant Alzheimer’s disease, Alzheimer’s disease and related dementias

## Abstract

Little is known about the characteristics and causes of early-onset cognitive impairment. Responders to the 2001 New York World Trade Center disaster represent an ageing population that was recently shown to have an excess prevalence of cognitive impairment. Neuroimaging and molecular data demonstrate that a subgroup of affected responders may have a unique form of parietal-dominant Alzheimer’s Disease. Recent neuropsychological testing and artificial intelligence approaches have emerged as methods that can be used to identify and monitor subtypes of cognitive impairment. We utilized data from World Trade Center responders participating in a health monitoring program and applied a deep learning approach to evaluate neuropsychological and neuroimaging data to generate a cortical atrophy risk score. We examined risk factors associated with the prevalence and incidence of high risk for brain atrophy in responders who are now at midlife. Training was conducted in a randomly selected two-thirds sample (*N* = 99) enrolled using of the results of a structural neuroimaging study. Testing accuracy was estimated for each training cycle in the remaining third subsample. After training was completed, the scoring methodology that was generated was applied to longitudinal data from 1441 World Trade Center responders. The artificial neural network provided accurate classifications of these responders in both the testing (Area Under the Receiver Operating Curve, 0.91) and validation samples (Area Under the Receiver Operating Curve, 0.87). At baseline and follow-up, responders identified as having a high risk of atrophy (*n *=* *378) showed poorer cognitive functioning, most notably in domains that included memory, throughput, and variability as compared to their counterparts at low risk for atrophy (*n *=* *1063). Factors associated with atrophy risk included older age [adjusted hazard ratio, 1.045 (95% confidence interval = 1.027–1.065)], increased duration of exposure at the WTC site [adjusted hazard ratio, 2.815 (1.781–4.449)], and a higher prevalence of post-traumatic stress disorder [aHR, 2.072 (1.408–3.050)]. High atrophy risk was associated with an increased risk of all-cause mortality [adjusted risk ratio, 3.19 (1.13–9.00)]. In sum, the high atrophy risk group displayed higher levels of previously identified risk factors and characteristics of cognitive impairment, including advanced age, symptoms of post-traumatic stress disorder, and prolonged duration of exposure to particulate matter. Thus, this study suggests that a high risk of brain atrophy may be accurately monitored using cognitive data.

## Teaser

Dementia has a rising major impact on the health of ageing populations. World Trade Center responders are at increased risk for dementia, as characterized particularly by parietal-dominant cortical atrophy on neuroimaging. Clouston et al. used deep learning to predict the risk of developing cortical atrophy based on neuropsychological test results.

## Graphical abstract

**Graphical Abstract:**
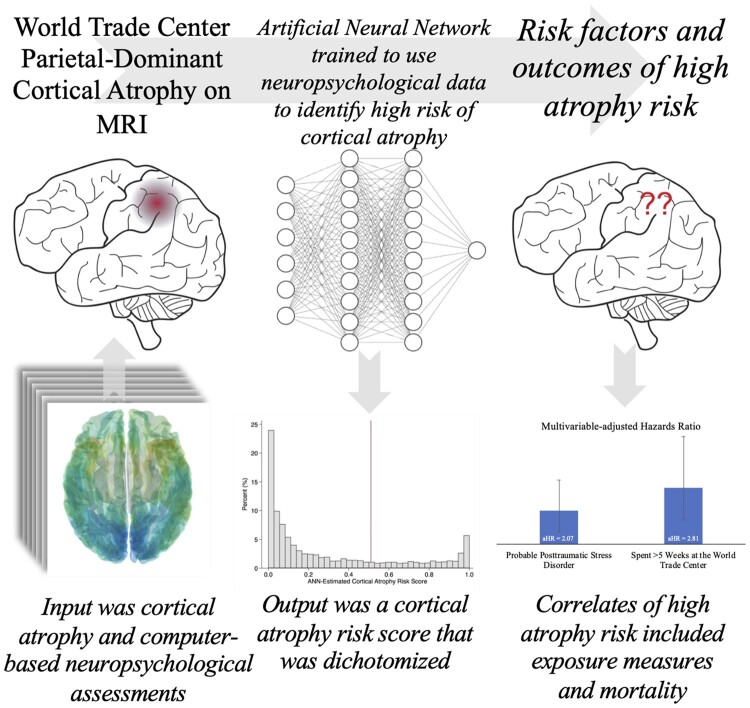

## Introduction

Alzheimer’s disease and related dementias (ADRDs) are collectively the cause of more than 100 000 deaths each year and represent a significant financial burden for affected individuals and their families, who often are called on to provide long-term care.^1^ADRDs are believed to result in specific cognitive changes throughout patients’ clinical course.^2^ Studies focussed on the general ageing population have revealed that ADRD represents a heterogeneous group of clinical entities that are influenced by a diverse set of environmental and cognitive modulators.^3^ A critical question in geriatric research is how to generate accurate diagnoses of new-onset age-related dementia to classify new ADRDs. This will be critical for investigating patient- and demographic-specific risk factors and developing effective treatments that will lead to improved patient care and management.

Responders to the World Trade Center (WTC) collapse have an excess risk for developing cognitive impairment. WTC responders have a unique set of characteristics that contribute to the observed increased risk of developing mild cognitive impairment (MCI).^4^ WTC responders were exposed to traumatic events and environmental factors that ultimately yielded various new psychiatric and medical diagnoses.^5^ WTC responders have a higher prevalence of post-traumatic stress disorder (PTSD),^6^ a risk factor for developing MCI in WTC responders.^7–9^ Additionally, WTC responders were exposed to debris that contained neurotoxic particulate matter (PM) that increased the risk of developing neuropsychiatric pathologies.^10^^,^^11^ In our recent longitudinal study, we found that prolonged WTC exposures increased the risk of MCI and, moreover, that the incidence of MCI was three times greater among the responders with prolonged exposure to the WTC site who also carried the apolipoprotein-ε4 allele (APOE-ε4) compared to those who did not carry this allele.^4^

There is evidence to suggest that neurodegeneration in the WTC population may present with unique characteristics compared to that experienced by the typical Alzheimer’s disease patient, which suggests a unique subtype of ADRD. For example, structural MRI studies have revealed that dementia in WTC responders is associated with marked cortical thinning that has characteristics like the recently identified parietal-dominant Alzheimer’s disease subtype.^12^ Similarly, one study focussed on plasma biomarkers identified the Alzheimer’s disease-associated Amyloid, Tau and Neurodegeneration neuropathological cascade among these responders.^11^ Previous literature suggested neuropsychological testing could help in monitoring the risk of developing ADRD, although it is unclear when to begin the methods that might be used to monitor cases of early-onset ADRDs, or potentially novel subtypes. Additional subdivisions and subtypes of ADRD based on neuropsychological assessments^13^ have been identified using machine learning techniques in patients with clinical ADRD findings used.^14^ These findings suggest that, in addition to an assessment of biomarkers, disentangling specific cognitive subtypes identified by performance on neuropsychological tests may be useful for non-invasive longitudinal tracking and for determining the phenotype for ADRD in the WTC responders.

Recent work has reported cortical atrophy in WTC responders with dementia,^12^ and a WTC-specific signature was developed to identify dementia with a high degree of accuracy.^15^ The objective of this study was to develop neuropsychological profiling tools that would facilitate large-scale non-invasive monitoring of the risk of developing cortical atrophy among these WTC responders. Our secondary goal was to examine demographic- and exposure-related correlates associated with high risk of atrophy (HAR) in a large cohort of WTC responders. We hypothesized that an artificial neural network (ANN) could be trained using neuropsychological testing profiling data to perform a reliable differentiation between responders with and without cortical atrophy observed by neuroimaging. Because the use of HAR to predict outcomes and its ongoing relationship to WTC-related exposure severity are both central to our study, we also attempted to understand the role of HAR in relation to specific epidemiologic variables. We hypothesized that responders who participated in an epidemiologic study of neuropsychological functioning, but who had no relevant neuroimaging findings, would present with HAR based on neuropsychological profiling. We found that this sub-grouping was associated with age, WTC exposure duration, symptoms of PTSD and with increased rates of mortality.

## Methods

### Setting

This study reports analyses from a cohort of WTC responders who were recruited from a prospective longitudinal study of cognitive performance of WTC responders that began in 2015 as previously described.^9^ Briefly, the parent study is a health surveillance program established by the Centers for Disease Control and Prevention that monitors 79 001 responders that had enrolled as of July 2020.^16^ Stony Brook University actively monitors WTC responders who mainly reside on Long Island in New York (NY) and facilitates research designed to identify disease aetiologies.

### Study design

**Figure 1:**
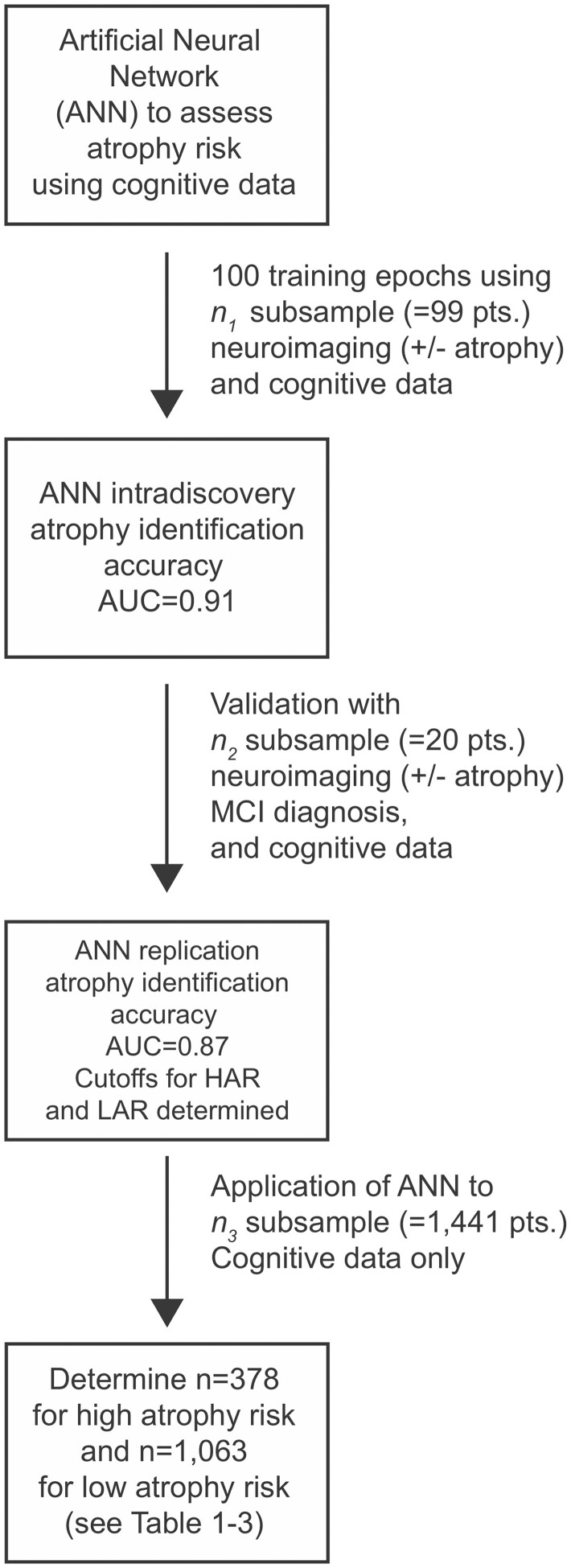
**Study flow diagram.** This flowchart diagram depicts the major steps in artificial neural network training and development of the atrophy risk score based on neuroimaging and cognitive data from World Trade Center responders as inputs. Three major steps were used to train an artificial neural network to assess atrophy risk based on the results of neuropsychological testing. Step 1: sample *n*_1_ included World Trade Center responders (participants; pts.) with neuroimaging data (±cortical atrophy) that was linked to results of neuropsychological testing. The artificial neural network underwent training to classify atrophy risk based on the associated cognitive tests. Step 2: *n*_2_ subsample with similar neuroimaging data was used to validate the artificial neural network trained in Step 1. Cutoffs were determined for high atrophy risk and low atrophy risk classifications. Step 3: We used the validated artificial neural network to study, classify and characterize the longitudinal dataset, *n*_3_.

We sought to develop a profiling metric developed *via* the use of an ANN that was based on the results of two neuroimaging studies of cortical atrophy because we are interested in the potential risk of developing one or more of the rare forms of ADRD, including parietal-dominant Alzheimer’s disease. We will refer to these studies as *n*_1_ and *n*_2_; see Fig. 1 for a description of the study flow. We relied on the larger of the two neuroimaging studies (*n*_1_) to serve as the discovery sample when training the ANN. We used the results from the second neuroimaging study (*n*_2_) to the validate the out-of-sample predictive accuracy of the scoring tool. Eligibility criteria for participating in these two neuroimaging studies have been previously described.^12^ Briefly, *n*_1_ and *n*_2_ included WTC responders 44–65 years of age who were fluent in English and had completed diagnostic assessments of WTC-related PTSD who were either cognitively normal or presenting with possible dementia in the discovery sample (*n*_1_ = 99) and MCI in the validation sample (*n*_2_ = 20). In both studies, cases were matched both demographically, in terms of PTSD, and occupationally to healthy, cognitively unimpaired WTC responders. Matching criteria included age, sex, race/ethnicity, education, geography and occupation held by each participant on 11 September 2001. Average age and demographics for both *n*_1_ and *n*_2_ matched the study population by design; for example, the mean age of those in the possible dementia discovery sample (*n*_1_) was 56.4 years versus 56.0 years for those in the validation sample (*n*_2_).

Next, we examined exposure-related correlates of HAR in a large cohort undergoing serial cognitive testing (*n*_3_) as previously described.^9^ We calculated HAR scores in a cognitive monitoring study that included up to four years of follow-up monitoring (follow-up response rate = 81.6%). This cognitive study followed a prospective design; responders who refused cognitive assessments at baseline remained eligible to participate in follow-up interviews; the final longitudinal analysis sample of *n*_3_ was 1441. Responders who presented with a baseline history of neurological diagnoses (e.g. multiple sclerosis, stroke, brain cancer, all-cause dementia) were excluded from further study or analysis along with those diagnosed with a WTC- or military-associated traumatic brain injury (*n* = 22). Study covariates associated with those who participated in the cognitive assessments did not differ from those of other eligible participants who did not undergo cognitive assessment, although we identified a trend suggesting that individuals of Hispanic origin were more likely to participate (*P *=* *0.016) than were Whites. Longitudinally, responders who were exposed to the WTC site for >5 weeks during the WTC search and rescue efforts were more likely to refuse follow-up cognitive testing compared to those who less exposed (*P *<* *0.001).

### Ethics

The Institutional Review Boards at both Stony Brook University and the Icahn School of Medicine at Mount Sinai approved the study procedures for *n*_1_. The Institutional Review Board at Stony Brook University alone approved the procedures for the other two studies. All participants provided written informed consent to participate in all research studies.

### Measures

#### Cognitive functioning

*Computer-Assessed Cognitive Performance* (CogState) is a 30-min computer-administered cognitive battery^17^ that measures fluid cognition using performance data and metadata in game-like tasks. Cognitive domains measured (Supplementary Table 1 has measurement-specific information) included reaction speed [i.e. Detection (DET) Task; ‘Has the card turned over?’], processing speed [i.e. Identification (IDN) Task; ‘Is the card red?’], intra-item response variability (i.e. IDN Task, measured as within-person variability in task responses), attention (i.e. IDN Task, measured in arcsine-transformed correct responses per tasks completed), visual memory [i.e. One Card Learning (OCL) Task; ‘Have you seen this card before?’], and throughput (i.e. OCL Task, measured as accuracy/speed). Earlier work with CogState in this population revealed that cognitive dysfunction in reaction speed, processing speed, visual memory, and throughput was associated with both long-term exposures to WTC sites and symptoms of severe and chronic PTSD.^9^

Clinical assessment of global cognitive functioning relied on the Montreal Cognitive Assessment^18^ following NIA-AA criteria^19^ with a score of ≤23 (/30 points) with evidence of cognitive decline being used to identify MCI^20^; amnestic MCI was determined using the memory impairment score.^21^ The label of possible dementia was provided following the NIA-AA criteria using a Montreal Cognitive Assessment ≤ 20 to indicate cognitive impairment consistent with dementia.^22^

#### Risk factors

Physical health risk factors were obtained from medical records and included those that have already been studied in cognitive impairment, such as hypertension,^23^ diabetes^24^ and cardiovascular disease.^25^ For this study, physical and clinical characteristics, including diagnoses of diabetes, hypertension and cardiovascular dysfunction were taken directly from patient histories. WTC exposure severity was assessed at the initial monitoring visit and the severity of exposure was defined as working >5 weeks with the WTC search and rescue efforts and sustaining physical injuries. Pulmonary exposure severity, a proxy of coarse-PM exposures, was assessed as proximally to the WTC exposures as possible, often as early as 2002, and characterized following prior guidelines.^26^

#### Developing the atrophy risk score and cutoff to identify HAR

The following describes the method of training, testing, and then examining longitudinal characteristics and development of an atrophy risk score using neurocognitive information (see Graphical Abstract for study flow and design). First, we applied an artificial neural network (ANN) protocol with four layers that included input and output layers. This was used to create a risk score based on the cognitive parameters revealed by the performance of the CogState tasks^27^ to identify cortical atrophy as assessed by MRI from the two aforementioned neuroimaging studies (*n*_1_ and *n*_2_)^12^; using an established cutoff.^15^

Training and testing for the ANN were done using the discovery sample (*n*_1_); randomized *k*-fold cross-validation was used to create the learning process. Randomization was used because it allowed us to examine the learning curves and to retrain the ANN in a way that we believed was more akin to human-based pedagogical and experiential learning. This also increased the number of times that the ANN could be trained. Because training requires more statistical power than testing, we apportioned a random sample consisting of approximately 66% of the data from *n*_1_ for training; the remaining 33% of the data from *n*_1_ were used to determine intra-discovery testing accuracy. Each ‘epoch’ consisted of 1000 training sessions; the order of cases was randomized within each subsample and the ANN underwent iterative training. Epochs were used to retrain and test the network to achieve a stronger identification of WTC-associated HAR using the CogState variables within the *n*_1_ sample portion only. The ANN completed 100 training epochs (each of which contained 1000 training sessions that were defined using a learning rate of 0.10). The goal for each session was to increase the accuracy in which cortical atrophy was predicted from data provided in the *n*_1_ sample.

After the learning component in the discovery sample from *n*_1_ was completed, the ANN was tasked to score cognitive data from the (separate) validation sample (*n*_2_ = 20), to assess whether the ANN could correctly sort responders that did or did not have evidence of cortical atrophy in the validation sample.

In both the discovery and validation samples, we measured accuracy by the area under the receiver operating curve (AUC) with corresponding 95% confidence intervals (CIs). The AUC show excellent (AUC = 0.90–1.00), strong (0.80–0.90), moderate (0.70–0.80), weak (0.60–0.70) or no predictive power (AUC = 0.50–0.60). Because the distribution of the WTC responders with HAR was bimodal, Youden’s method was used to determine conservative cutoffs for the validation sample (*n*_2_) for the best performing model. These cutoffs were used to categorize outcomes for analyses across all samples (both *n*_1_ and *n*_2_). The ANN was generated using the Brain package^27^; included in the Appendix Neural Network File 1. The final analyses of the risk score and cutoffs were used to evaluate the population in the monitoring cohort, *n*_3_, as described above.

### Statistical analyses

Descriptive statistics for the study were provided for the sample at baseline and for those separated into responders associated with the two groups identified by the ANN, the low atrophy risk (LAR) and HAR groups. Means and standard deviations were reported for continuous variables; percentages were reported for categorical variables. Bivariable *P*-values were used to examine differences between responders with LAR and HAR using χ^2^ tests for categorical variables and Student’s *t*-tests for continuous variables.

Cross-sectional analyses of HAR risk at enrolment in the monitoring cohort *n*_3_ relied on multivariable log-binomial regression to estimate unadjusted and adjusted risk ratios. The first model incorporated PTSD and adjusted solely for demographics while the second model also incorporated three measures of WTC exposure severity; earlier studies have consistently identified only WTC exposure duration as relevant to neurocognitive functioning. The full model also adjusted for common risk factors, including cardiovascular disease and diabetes.

Longitudinal multivariable analyses used Cox proportional hazards regression^28^ to estimate the hazards associated with the incidence of HAR. Incidence of HAR as identified by the ANN was the outcome, and individuals were censored at final follow-up or at time of death. Multivariable-adjusted hazards ratios (aHRs) were reported. The proportional hazards assumption was examined using Schoenfeld residuals; all models passed this assumption. The population attributable fraction was calculated using aHRs from the longitudinal model.^29^

To validate the HAR against clinical indicators associated with increased risk, we also examined predictions of clinical outcomes with diagnoses that included amnestic MCI, multidomain MCI, and dementia. For exploratory purposes, and to examine the impact of possible missing data due to mortality, we also used this approach to analyse the relationship between the HAR and mortality (*n* = 18 died among in *n*_3_) while adjusting for both age and sex.

The current study represents a secondary data analysis; the power needed to detect outcomes was not determined specifically for this study. However, post-hoc power analyses revealed that the power to detect effect sizes included >7% differences in relative risk with a power = 0.80 using a two-tailed *α* = 0.05.

All analyses were performed using Stata 16/SE (StataCorp) and the ANN protocol was implemented using the Brain package.^27^

### Data availability

Medical diagnoses and dates of diagnosis remain the private health information. of each participant. Therefore, access to data and code for data analysis will be provided upon receipt of a written request by the corresponding author.

## Results

### Step 1: Developing and validating HAR

In the first experiment, we trained an ANN to predict cortical atrophy (i.e. an atrophy risk score) as identified on neuroimaging studies using CogState scores as the input. From these outcomes, we obtained a classification accuracy for training with excellent accuracy in the intra-discovery accuracy testing sample, *n*_1_ (AUC, 0.91; 95% CI 0.83–0.98). The learning curve is shown in Supplementary Fig. 1. Output associated with CogState variables used by the ANN in its prediction efforts (Supplementary Table 2) revealed that visual memory and high response variability as indexed by CogState were central features of the atrophy risk score and achieved strong results in the validation sample, *n*_2_ (AUC, 0.87; 95% CI, 0.70–1.00). Youden’s method for determining the optimal cut-off point for HAR includes risk scores ≥0.51 in the validation sample. Thus, these findings validated the ability of the ANN to identify cortical atrophy in WTC responders.

### Step 2: Results from epidemiologic findings that implement the HAR scoring methods

**Figure 2:**
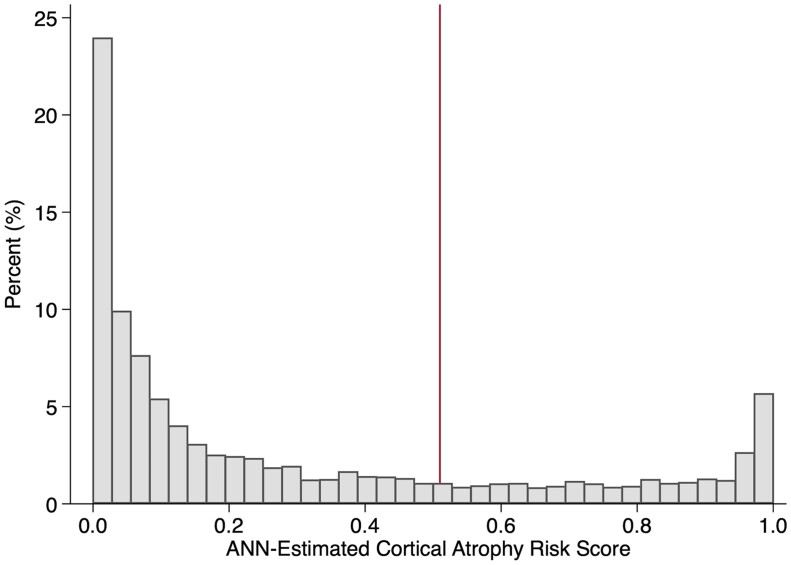
**Cortical atrophy risk score distribution in World Trade Center responders.** The distribution of Cortical Atrophy Risk scores indicating increasing risk of cortical atrophy as estimated in the longitudinal follow-up study of World Trade Center responders (*n*_3_). A bar histogram of the distribution of atrophy risk scores and the percentage of each score identified in the longitudinal sample (*n*_3_). The vertical red line shows the cutoff for high atrophy risk.

The findings in Table 1 include the summarized CogState results and characteristics relevant to the entire sample used for the two ANN-determined groups, i.e. LAR and HAR. In Fig. 2, we plotted the distribution of risk scores in the epidemiologic sample (*n*_3_); these findings ranged from low (0) to high cognitive functioning (1.0) and had a mean of 0.704 [standard deviation (SD = 0.332)]. The HAR group displayed cognitive scores that were similar to those of the training sample that included WTC participants with neuroimaging findings indicative of cognitive dysfunction and cortical atrophy; these findings were not associated with pulmonary exposure severity when compared to LAR (mean, 4.97; SD, 5.17 for HAR versus mean, 5.20; SD 5.11 for LAR, *P *=* *0.468).

**Table 1:** Descriptive sample characteristics at baseline and separated by atrophy risk. Cognitive domains and characteristics of the epidemiological sample (*n*_3_) overall and separated into low and high atrophy risk cohorts at baseline

	Epidemiologic Sample at first cognitive assessment (*n*_3_) (*N* = 1441)	Low atrophy risk at baseline (LAR) (*n* = 1063)	High risk of cortical atrophy at baseline (HAR) (*n* = 378)	
Cognitive characteristics	Mean (SD)	Mean (SD)	Mean (SD)	*P*
Cognitive domains				
Visual memory	0.98 (0.10)	0.99 (0.09)	0.93 (0.09)	<1E-06
Throughput	0.33 (0.03)	0.33 (0.03)	0.30 (0.03)	<1E-06
Intra-individual variability	0.08 (0.03)	0.07 (0.02)	0.11 (0.03)	<1E-06
Processing speed	0.08 (0.01)	0.08 (0.01)	0.07 (0.01)	<1E-06
Reaction speed	0.07 (0.01)	0.07 (0.00)	0.06 (0.01)	<1E-06
Attention	1.42 (0.14)	1.42 (0.13)	1.41 (0.17)	0.072
Demographic and clinical characteristics				
Age in years	53.53 (8.31)	52.37 (7.86)	56.78 (8.67)	<1E-06
Pulmonary exposure severity	5.03 (5.15)	4.97 (5.17)	5.2 (5.11)	0.468
Posttraumatic stress disorder symptomatology				
Re-experiencing	0.29 (0.35)	0.27 (0.33)	0.35 (0.40)	7.33E-05
Avoidance	0.30 (0.46)	0.28 (0.44)	0.35 (0.49)	0.011
Numbing	0.22 (0.35)	0.20 (0.32)	0.28 (0.40)	3.90E-05
Hyperarousal	0.39 (0.44)	0.36 (0.42)	0.46 (0.49)	1.95E-04
Female sex, %	7.70	7.06	9.52	0.122
Race/Ethnicity, %				
White	87.65	88.81	84.39	0.021
Black	2.50	1.79	4.50	
Other	1.32	1.22	1.59	
Hispanic	8.54	8.18	9.52	
Hypertension, %	29.29	25.78	39.15	<1E-06
Diabetes, %	8.74	6.87	14.02	2.35E-05
Cardiovascular disease, %	11.59	10.44	14.81	0.023
Injured at the WTC site, %	16.38	16.56	15.87	0.758
Spent >5 weeks on-site, %	50.45	49.11	54.23	0.087
Probable post-traumatic stress disorder	11.80	9.97	16.93	3.15E-04

Cognitive domains measured for this study included reaction speed [i.e. Detection (DET) Task, ‘Has the card turned over?’ measured as answers per second; a higher score is better than a lower score]; processing speed [i.e. Identification (IDN) Task, ‘Is the card red?’ measured as answers per second; a higher score is better than a lower score], intra-item response variability (IDN Task, measured as SD; a lower score is better than a higher score), attention (IDN Task, measured as the accuracy of performance; a higher score is better than a lower score), visual memory [One Card Learning (OCL) Task, ‘Have you seen this card before?’ measured as the accuracy of performance; a higher score is better than a lower score], and throughput (OCL Task, measured as accuracy/speed); *P*-values were derived from t-tests for continuous variables or chi-square tests for categorical variables.

WTC, World Trade Center.

### Bivariate correlates of WTC-HAR

WTC responders in the HAR group were older than those in the LAR group (mean difference, 4.41 years; 95% CI 3.46–5.36; *P *<* *1E-06). As might be anticipated, responders in the HAR group achieved lower scores across most of the categories when compared to those in the LAR group, including those involving visual memory (mean, 0.99; SD, 0.09 versus mean 0.93; SD, 0.09; *P *<* *1E-06), throughput (mean, 0.33; SD, 0.03 versus mean 0.30; SD, 0.03; *P *<* *1E-06), intra-individual response variability (mean, 0.07; SD, 0.02 versus mean 0.11; SD, 0.03; *P* < 1E-06,), processing speed (mean, 0.08; SD, 0.01 versus mean, 0.07; SD, 0.01; *P *<* *1E-06), and reaction speed (mean, 0.07; SD, 0.00 versus mean, 0.06: SD, 0.01; *P *<* *1E-06). However, we detected no significant differences between outcomes of the HAR group versus those of the LAR group for the attention variable (*P *=* *0.072).

We then determined whether demographic and clinical factors, including those previously implicated in cognitive dysfunction associated with ageing, might be associated with HAR. WTC responders in the HAR group displayed higher levels of PTSD symptomatology compared to those in the LAR group, including re-experiencing (mean, 0.27; SD, 0.33 versus mean 0.35; SD, 0.40; *P *=* *7.33E-05), avoidance (mean, 0.28; SD, 0.44 versus mean, 0.35; SD, 0.49; *P = *0.011), numbing (mean, 0.20; SD, 0.32 versus mean, 0.28; SD, 0.40; *P = *3.90E-05) and hyperarousal (mean, 0.36; SD, 0.42 versus mean, 0.46; SD, 0.49; *P = *1.95E-04). There was a significantly higher proportion of older versus younger responders, as well as females, and those of non-Whites in the HAR compared to LAR (see Table 1). Additionally, WTC participants with HAR were more likely to be diagnosed with comorbidities, including hypertension (39.15% versus 25.78%, *P *<* *1E-06), diabetes (14.02% versus 6.87%, *P *=* *2.35E-05), and cardiovascular disease (14.81% versus 10.44%, *P* = 0.023).

### Multivariable-adjusted correlates of the HAR prevalence among WTC responders

The prevalence of HAR at initial cognitive assessment was estimated to be 26.23% (95% CI 23.98–28.58%). In Table 2, we examined WTC-related risk factors associated with the ANN neuropsychological signature and found that age, PTSD, and race/ethnicity were all associated with HAR at the initial assessment. Interestingly, each additional year of age was associated with a 4.2% increased risk of HAR among those in this population. Of note, those with probable PTSD had a higher risk of HAR at baseline. Furthermore, results also revealed a trend towards higher risk among participants who spent >5 weeks on-site at the WTC. Secondary results include a higher risk among those diagnosed with hypertension and heightened risk in Black responders across all models.

**Table 2:** Analyses of prevalence of high atrophy risk. Risk ratios for baseline factors in relation to high atrophy risk among World Trade Center responders

Characteristics	Demographically adjusted model			Multivariable-adjusted model		
	aRR	95% CI	*P*	aRR	95% CI	*P*
Age in years	1.046	1.034–1.058	0.000	1.042	1.029–1.055	0.000
Female sex	1.325	0.934–1.879	0.115	1.376	0.968–1.958	0.075
Race/Ethnicity						
White	1.000			1.000		
Black	2.000	1.221–3.276	0.006	1.940	1.181–3.186	0.009
Other	1.243	0.551–2.801	0.600	1.160	0.513–2.624	0.721
Hispanic	1.182	0.836–1.672	0.343	1.196	0.844–1.694	0.315
Probable post-traumatic stress disorder	1.475	1.126–1.934	0.005	1.481	1.127–1.948	0.005
Pulmonary exposure severity				1.000	0.98–1.019	0.976
Injured at the WTC site				0.847	0.639–1.123	0.249
Spent >5 weeks on-site				1.224	0.995–1.504	0.055
Hypertension				1.256	1.003–1.572	0.047
Diabetes				1.190	0.874–1.621	0.270
Cardiovascular disease				0.981	0.726–1.325	0.898

Adjusted risk ratios were derived from log-binomial relative risk models.

CI, confidence interval; aRR, multivariable-adjusted risk-ratio; WTC, World Trade Center.

### Multivariable-adjusted correlates of incidence of WTC-HAR

The incidence of HAR in this sample was estimated at 10.02 events per 100 person-years (95% CI 8.67–11.58). In total, we identified 183 cases of HAR among those who presented as LAR at baseline and who participated in at least one follow-up assessment (*n* = 960). Each year of age was associated with a 4.5% increase in the incidence of HAR in this population. In our examination of the hazards associated with the incidence of HAR (Table 3), we found individuals diagnosed with probable PTSD who spent >5 weeks at the WTC site had a greater aHR across all models, including both the Exposure Adjusted Model (aHR, 2.816; 95% CI 1.782–4.451; *P *<* *0.001) and the Multivariable Adjusted Model (aHR, 2.815; 95% CI 1.781–4.449; *P *<* *0.001). The population attributable fractions were estimated to be 21.9% for probable PTSD and 32.3% for spending >5 weeks at the WTC site. By contrast, we found a protective effect among those with high reported pulmonary exposure severity that occurred primarily during the immediate aftermath of the WTC collapse. Secondarily, we found trends towards a higher incidence of HAR among Black responders, and a significantly higher incidence of HAR among Hispanic responders compared to responders identified as White.

**Table 3:** Analyses of incidence of high atrophy risk. Multivariable-adjusted hazards ratios (aHRs) examining World Trade Center exposures and post-traumatic stress disorder as risk factors for the incidence of high atrophy risk at follow-up among World Trade Center responders identified as cognitively unaffected at baseline

Characteristics	Demographically adjusted model			Multivariable-adjusted model		
	aHR	95% CI	*P*	aHR	95% CI	*P*
Age, years	1.047	1.029–1.065	<0.001	1.045	1.027–1.065	<0.001
Female sex	1.090	0.583–2.037	0.787	1.209	0.642–2.275	0.557
Race/ethnicity						
White	1.000			1.000		
Black	2.282	0.926–5.624	0.073	2.325	0.923–5.855	0.074
Other	0.714	0.333–1.533	0.388	0.710	0.330–1.527	0.381
Hispanic	3.360	1.671–6.755	0.001	2.477	1.216–5.046	0.012
Probable post-traumatic stress disorder	2.153	1.475–3.142	<0.001	2.072	1.408–3.050	<0.001
Pulmonary exposure severity				0.958	0.929–0.987	0.005
Injured at the WTC site				1.087	0.746–1.584	0.664
>5 weeks on-site				2.815	1.781–4.449	<0.001
Hypertension				1.119	0.799–1.566	0.514
Diabetes				1.083	0.646–1.818	0.762
Cardiovascular disorders				1.360	0.922–2.006	0.121

Adjusted risk ratios were derived from a log-binomial relative risk model.

CI, confidence interval; aHR, multivariable-adjusted hazards ratio; WTC, World Trade Center.

### Indicators of clinical severity

On average, 28.2% and 20.7% of the participants identified as HAR had amnestic or multidomain MCI, compared to 12.2% (age/sex aRR, 2.06; 95% CI 1.74–2.42; *P *<* *1E-06) and 7.4% (aRR, 2.33; 95% CI 1.86–2.91; *P *<* *1E-06) of those with LAR, respectively. Similarly, 5.7% of those identified as HAR had dementia versus 1.8% of those in the LAR group (aRR, 2.41, 95% CI 1.55–3.74; *P *<* *1E-06). Of the 16 responders who died during the observation period, 10 had features consistent with HAR; interestingly, only six of the participants assigned to the LAR group indicating that HAR responders were at increased risk of mortlaity as comopared to LAR responders (RR, 4.51; 95% CI 1.65–12.32; *P *=* *0.003). This result remained statistically significant after adjusting for both age and gender (aRR, 3.19; 95% CI 1.13–9.00; *P *=* *0.029).

## Discussion

ADRD represents a major public health concern worldwide that may also be important to consider in the WTC responder population.^4^^,^^9^^,^^30^ Findings reported in previous studies revealed that neuroimaging signatures could reliably identify cortical atrophy consistent with parietal-dominant Alzheimer’s disease in WTC responders.^12^ Combined with artificial intelligence, neuropsychological testing represents a standard non-invasive method that might help to diagnose and monitor different subtypes of ADRD.^14^^,^^31^ Prior work has focussed on results encompassing multiple domains of cognition and as a result, it is not clear whether a WTC responder is at high risk for cortical atrophy as determined by neuroimaging. This study is the first to address this gap and it also validates a non-invasive method that identifies responders at high risk for developing cortical atrophy at midlife.

This study uniquely sought to determine whether a deep learning approach applied to neuropsychological testing, that included imaging biomarkers of ADRD, could be used to develop a cognitive atrophy risk score to monitor the neurological status of ageing WTC responders. We hypothesized that the WTC responders who developed ADRD would have distinct neuropsychological testing deficits. Accordingly, we hypothesized that an ANN could be trained to recognize neuropsychological testing deficits using test results from WTC responders who had neuroimaging biomarkers consistent with ADRD (i.e. parietal-dominant cortical thinning). Our ANN classification identified a group in which a decline in cognitive scores was associated with a constellation of factors including female sex, specific race/ethnicity, and cardiovascular co-morbidities. These results suggest that decreases in neuropsychological testing scores associated with demographic risk factors may result in an increased likelihood of cortical atrophy. Below, we discuss our results as they relate to our current understanding of neuropsychological testing, parietal-dominant Alzheimer’s disease, WTC-related exposures and demographic factors.

Many researchers have used neuropsychological testing at midlife to distinguish the different neurodegenerative subtypes.^14^^,^^32^^,^^33^ These efforts have achieved 89% accuracy for the identification of late-onset Alzheimer’s disease,^33^ and 92–96% when using a similar approach to identify late-onset Alzheimer’s disease in a large hospital-based geriatrics assessment program.^14^ Our results were able to reliably identify individuals with evidence of cortical atrophy (AUC, 0.87; 95% CI 0.70–1.00). The accuracy of our program was notably high given the relatively small sample size. Further work will be needed, including MRI investigations in larger cohorts, to refine the ANN and improve predictions as necessary.

We were unable to determine a unique domain for cognitive impairment that identified HAR using the CogState variables. Instead, we needed to rely on a combination of these measures. Previous work revealed that, in comparison to more typical disease presentations, parietal-dominant Alzheimer’s disease is associated primarily with deficits in executive functioning, including working memory, task switching, inhibition, and visuospatial processing.^34^ Moreover, there has been much recent interest in understanding the role of practice effects with repeated testing as a means to differentiate ADRDs from ordinary ageing with individuals by focussing on patients with early-stage Alzheimer’s disease who did not reliably exhibit practice-related improvement over time.^35^ However, the extent to which these observations remain true for individuals exhibiting parietal-dominant Alzheimer’s disease is not known. Nevertheless, results from a recent study revealed that cognitive variability as measured by intra-item response variability is common in otherwise cognitively normal individuals with early Alzheimer’s disease,^36^ indicating that this phenotype may be a critical feature associated with the disease process. The clinical impact of cognitive variability may result in unreliable characterizations with the potential to masquerade as long-term improvement in cognitive function. Future work on this subject might consider the inclusion of more sensitive measures of executive functioning and visuospatial processing in the cognitive testing battery and may also consider the impact of repeated testing for characterizing HAR.

Parietal-dominant Alzheimer’s disease is a recently described subtype that appears to be more common in relatively younger populations (mean age 60.4 years^37^) like the disorder experienced by the WTC responder population described here. Among the differences between these subtypes, parietal-dominant Alzheimer’s disease has been associated with reduced episodic memory, working memory, and executive functioning. Furthermore, individuals diagnosed with parietal-dominant Alzheimer’s disease experienced a more rapid progression of the disease. Notably, in this subtype, cognitive decline is consistent with a more aggressive subtype of Alzheimer’s disease compared to symptoms characteristic of the more common late-onset form of the disease.^38^ In this study, we also found that the HAR designation was predictive of all-cause mortality; this finding may suggest that this condition heralds potentially severe disease. A notable strength of this study was use of cognitive variability as an essential characteristic of parietal-dominant Alzheimer’s disease. While further analyses will be needed to clarify the rate of progression to dementia among individuals in this population, our study allowed us to identify specific risk factors associated with HAR and thus further characterize the clinical dimensions of cognitive dysfunction experienced by the WTC responders.

Our neuropsychological profiling of the HAR cohort is also consistent with exposure of the WTC responder cohort to environmental risk factors. Exposure to fine and coarse PM has been identified as a potentially modifiable cause of ADRD.^39^ Researchers have identified associations between PM exposure and cortical atrophy in the temporal lobe^40^ and increased risk of clinical phenotypes, including symptomatology, proteinopathy, and neurodegeneration similar to that observed in Alzheimer’s disease.^41–43^ WTC responders represent a critical population to study in this regard because they were exposed to toxic elements that include strontium, barium, lead, aluminium, thallium, silicon, and manganese, among others.^44^ Many have also experienced chronic exposure to finer PM and polycyclic aromatic hydrocarbons.^45^^,^^46^ Our results are consistent with these findings; for example, reporting that incidence of HAR was lower in WTC responders with higher pulmonary exposures but elevated in those who spent long periods of time (>5 weeks) at the site of the WTC collapse. There is a substantial amount of evidence documenting the impact of exposure to and inhalation of fine PM. For example, PM_2.5_, ultra-fine PM and nano-PM can enter the brain through the olfactory epithelium and infiltrate into the circulation;^47^ this can disrupt the blood–brain barrier and may elevate neuroinflammation due to oxidative stress and neurotoxicity. While it is challenging to measure the specific PM amounts and content of PM exposure, we speculate that inhalation of PM at the site of the WTC collapse may accelerate brain ageing and contribute to MCI observed in the HAR group.

In addition to PM exposure, WTC-related chronic PTSD is another important risk factor uniquely prevalent in this population. The high prevalence of PTSD among the WTC responders is related to their experiences in severe and traumatic events.^6–9^^,^^48^^,^^49^ PTSD may be augmenting the impact of HAR and neuropathology related to PM exposure. Re-experiencing traumatic events can conjure up mental imagery and result in depersonalization^50^ and dissociation^51^; this may result in over-activation of allocortical and neocortical regions, including the hippocampus, limbic system, and the occipital lobes.^50–53^ In turn, this over-activation may contribute to neurodegeneration through mechanisms associated with neuroinflammation.^54^^,^^55^ For example, a recent report that examined cognitive functional limitations that were self-reported by a cohort of New York Fire Department (FDNY)-based WTC-responders highlighted the fact that PTSD may be a major contributor to cognitive limitations reported in this population.^56^ More comprehensive and objective neuropsychological batteries that do not rely on subjective, self-reported measures will be required to clarify cognitive dysfunction and atrophy risk in this population.

Given the predictive validity of the ANN identifications for this cohort of WTC responders, we determined whether the HAR group had other characteristics or demographics that might contribute to the development of MCI and dementia. Strikingly, we found other risk factors for HAR were a high prevalence of specific races/ethnicities and cardiovascular problems. This finding is consistent with those reported in earlier studies that investigated the relationships between metabolic health and the impact of sex and race on ageing.^57^ This provides further evidence for the convergent validity of the ANN identification generated here. Given the heterogeneity of presentation among individuals with MCI, it has become clear that an increased focus on multiple demographic factors may yield improved characterization of cognitive dysfunction and indicate progression and risk in the WTC responder population.

Our findings revealed an increased prevalence of HAR in Black WTC responders. This may be consistent with studies that document a higher incidence of Alzheimer’s disease in Black Americans.^58^ While the mechanisms underlying the impact of race/ethnicity on the processes underlying cognitive decline and risk of dementia with ageing remain unclear, several studies point towards the contributions of cardiovascular comorbidities, diet, socioeconomic disparities, and psychosocial stress.^30^^,^^59^ From a genetic standpoint, Black Americans are more likely to carry the APOE4-ε4 allele than are White Americans. This is a major genetic risk factor for Alzheimer’s disease that was also identified as an effect modifier for the impact of WTC exposure in this population.^4^ However, this finding does not explain the heightened risk in Hispanics in the WTC cohort, who are much less likely to be carrying the potentially harmful APOE-ε4 allele. Additional studies should be conducted to determine potential underlying genetic causes and subsequent mechanistic outcomes associated with race in this cohort.

### Strengths and limitations

Our study demonstrates that AI can classify cognitive dysfunction in a population at high risk for cortical atrophy. The utility of this function was additionally demonstrated by relationships between demographics and clinical factors that may contribute to the development of MCI and dementia. The WTC responder cohort includes primarily white males; this limits our ability to generalize responses to women and racial/ethnic minorities. Another limitation of this study is that the ANN was not trained to monitor or make predictions based on changes in the cognitive scores. Unfortunately, there are no longitudinal studies of cortical atrophy in this population, although this would be an important, natural extension of the current work. Further follow-up testing of the HAR group, and a broader population-based neuroimaging study might improve the longitudinal characterization and provide further improvements in the accuracy of the ANN. Previous studies have focussed on changes in cognitive scores as a function of time, which is a cornerstone of ageing research.^60^ Future studies using this analysis will determine whether our findings are helpful for following the progression of dementia or predicting the development of cortical atrophy among currently healthy WTC responders. It might also be fruitful to perform a longitudinal investigation of the WTC responders with HAR as determined by the ANN as they change over time, given the potential for ongoing cognitive decline identified in this cohort. Furthermore, while atrophy on MRI indicates neurodegenerative disease, according to the Alzheimer’s disease research framework,^2^ results should be validated with molecular imaging studies and cerebrovascular or plasma-based biomarkers. Numerous studies highlight disparities between clinical cognitive deficits and molecular neuropathological findings.^61^ Future studies will be needed to address the extent to which attributes contributing to HAR are true risk factors or whether this state is a stand-alone clinical entity that develops at midlife.

## Conclusion

To date, there is limited evidence focussed on the clinical progression of parietal-dominant Alzheimer’s disease at midlife. Likewise, no evidence exists that can be used to identify individuals at risk of developing a neurodegenerative disease. In the case of WTC responders, such evidence would perform effective monitoring of men and women at risk for WTC-related neurocognitive disorders. For the first time, this study provides a method that can be used to assess risk of developing cortical atrophy at midlife in a cohort of WTC responders, and may additionally perform to identify those with early parietal-dominant Alzheimer’s disease. Specifically, this study used an ANN-based risk profiling strategy to identify several risk factors, including PTSD and prolonged WTC longer-duration exposures collapse site, which were both statistically important predictors, over and above other established demographic and medical risk factors. Finally, preliminary work revealed that the designation HAR was associated with an increased risk of all-cause mortality. This work supports ongoing research and increasing evidence that cognitive functioning may be adversely affected in the WTC responder cohort and that this may be the result of occupational exposures experienced following the attacks of 11 September 2001.

## Supplementary material

Supplementary material is available at *Brain Communications* online.

## Funding

This study was funded by the National Institute on Aging (R01 AG049953), and the Centers for Disease Control and Prevention (200-2011-39361, and U01 OH011314).

## Competing interests

The authors report no competing interests.

## Supplementary Material

fcab145_Supplementary_Data

## Abbreviations

ADRDs =: Alzheimer’s Disease and related dementias
aHRs =: adjusted hazards ratios
ANN =: artificial neural network
HAR =: high atrophy risk
LAR =: low atrophy risk
MCI =: mild cognitive impairment
OCL =: one-card learning
PM =: particulate matter
PTSD =: post-traumatic stress disorder
WTC =: World Trade Center
