# Supplementary material for: A deep learning approach for monitoring parietal-dominant Alzheimer’s disease in World Trade Center responders at midlife
Source: Brain Commun. 2021 Jul 2;3(3):fcab145. doi: 10.1093/braincomms/fcab145 (PMC8361422; doi:10.1093/braincomms/fcab145)
Supplement: fcab145_Supplementary_Data [file fcab145_supplementary_data.zip › Supplementary_material Appendix table.docx]

**Appendix Table:** Brain Output Matrices

|  | Input | | | |  | Output |  | Flatline |
| --- | --- | --- | --- | --- | --- | --- | --- | --- |
|  | Minimum | Normalized | Value | Signal |  | Weight |  | Weight |
| Intra-Item Variability | 0.04 | 5.26 | 0.23 | 1.00 |  | 10.42 |  | 0.0097 |
| Response Speed | 0.05 | 21.85 | 0.09 | 1.00 |  | -5.41 |  | -0.6773 |
| Processing Speed | 0.04 | 32.76 | 0.07 | 1.00 |  | 0.58 |  | 0.0042 |
| Attention | 0.43 | 0.88 | 1.57 | 1.00 |  | -1.51 |  | -0.0327 |
| Visual Memory | 0.65 | 1.36 | 1.39 | 1.00 |  | 8.81 |  | 0.0097 |
| Throughput | 0.23 | 3.69 | 0.50 | 1.00 |  | -20.96 |  | -0.9903 |
|  |  |  |  |  |  |  |  |  |
| Output | 0.00 | 1.00 | 0.99 | 0.99 |  | 4.62 |  | 0.9903 |
